# Associations between body composition and physical fitness among Chinese medical students: a cross-sectional study

**DOI:** 10.1186/s12889-022-14548-0

**Published:** 2022-11-08

**Authors:** Bing Li, Lu Sun, Ye Yu, Hong Xin, Han Zhang, Jie Liu, Zhuo Zhang

**Affiliations:** 1 Department of Dermatology, Shengjing Hospital of China Medical University, Shenyang, 110004 China; 2 Radiation Health Center, Liaoning Provincial Center for Disease Control and Prevention, Shenyang, 110015 China; 3 School of Public Health, Shenyang Medical College, Shenyang, 110034 China; 4 Department of Physical Education, Shenyang Medical College, Shenyang, 110034 China

**Keywords:** Body composition, Physical fitness, Medical students

## Abstract

**Background:**

This study examined associations between body composition and physical fitness scores among medical students in Shenyang, China.

**Methods:**

A total of 2291 medical students aged 18–20 (815 male and 1476 female) in Shenyang of China were recruited to participate in the research in May 2019. With the use of the BCA-1B body composition analyzer and standard method of physical fitness assessment, the body composition and seven measures of physical fitness (body mass index, vital capacity, sit and reach, standing long jump, pull-ups/crunches, 50-m sprint, and 800/1000-m run) of college students were measured, respectively. The associations between body composition and physical fitness scores were assessed using multiple linear regression analysis.

**Results:**

The height, weight, total body water, protein mass, mineral content, body mass index, vital capacity, and body function scores of male students were significantly higher than those of female students. However, fat mass (FM), body shape score, physical quality score, and total physical fitness score of female students were significantly higher than those of male students. The results of the multiple linear regression analysis indicated that in male students, only FM was negatively associated with body shape score, body function score, physical quality score, and total physical fitness score. In female students, FM was associated with lower body shape scores, physical quality scores, and total physical fitness scores. Furthermore, the ratio of overweight to obesity in male students was significantly higher than that in female students.

**Conclusions:**

In Chinese medical colleges, the physical fitness level of female students is better than that of male students, and a higher FM was significantly associated with worse physical fitness of medical students. Moreover, male students with a higher rate of overweight and obesity are an important group that needs weight control.

## Background

In recent years, with the development of the social economy, physical fitness has become one of the most popular topics for research and discussion [1]. Previous research has shown that poor physical fitness can increase the risk of multiple diseases, such as cardiovascular disease and diabetes etc. [2]. Although China’s national physical fitness monitoring results indicate that the overall level of the country’s national physical fitness has been improving, the physical fitness status of college students is not optimistic, mainly due the changes in body shape and the deterioration of physical function and physical quality [3]. In Chinese physical fitness monitoring system, body shape refers to the external shape of an individual’s body, and physical function mainly represents the level of individual vital capacity, while physical quality is the feedback of the comprehensive level of human endurance, explosive power and flexibility. In particular, due to the tight schedule and heavy workload, the stress level of medical students is high, and the time that students spend on outdoor activities is relatively insufficient, resulting in their physical fitness levels decline shortly after entering medical college [4, 5]. As the reserve force and main force for the sustainable development of medical and health field, as well as the advocates of health promotion in the future, it is particularly important to focus on the physical fitness of medical students. In addition, it is found that the distribution of human body composition are closely related to the fitness status of college students and can also be used as an important index to evaluate the fitness status of college students [6–9]. However, in existing research data, there are few reports about the correlation between body composition and various indicators reflecting physical fitness status among medical students [10, 11]. To provide information to further improve the physical fitness of medical students, the aim of the study was to examine the gender characteristics of this group’s body composition and physical health status and to study the relationship between body composition and body shape, body function, and physical quality.

## Methods

### Participants

A total of 2340 medical students aged 18–20 years were recruited from Shenyang Medical College after the approval of the Ethics Committee of Shenyang Medical College (reference no. 2019–006) was obtained. Of the total sample population, 37 refused to participate in the current study. Moreover, eight individuals with respiratory, cardiovascular, and/or metabolic diseases such as diabetes and thyroid disease in recent years were excluded. In addition, data on anthropometric measurements of one male and three female students were missing owing to the students’ absence during data collection.

**Fig. 1:**
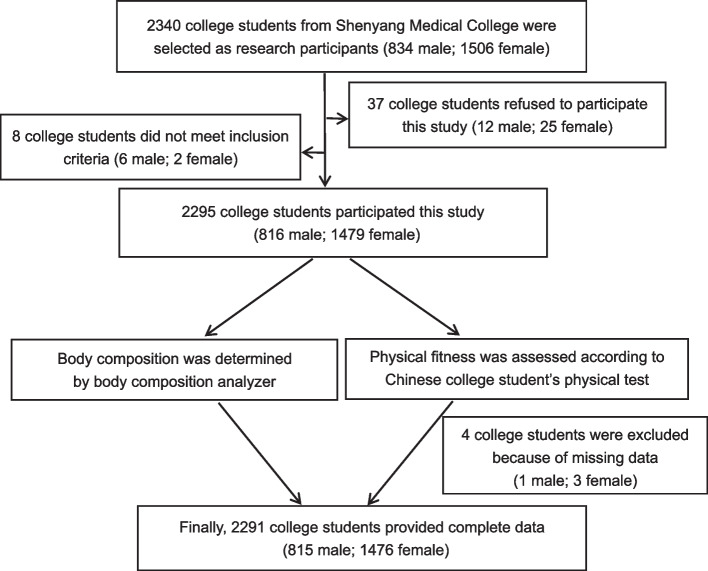
Flow chart of the study enrollment

Therefore, 2291 participants (19.09 ± 0.82 years old) in total (815 male and 1476 female) were eventually eligible for the statistical analyses, of whom 1916 (83.63%) were Han Chinese (708 male and 1208 female) and 375 (16.37%) were ethnic minorities: 92 Mongolians (30 male and 62 female), 13 Huis (5 male and 8 female), 12 Miaos (7 male and 5 female), 3 Yis (2 male and 1 female), 7 Zhuangs (2 male and 5 female), 6 Buyis (3 male and 3 female), 4 Koreans (2 male and 2 female), 220 Manchus (52 male and 168 female), 2 Dongs (1 male and 1 female), 1 Bai (female), 7 Tujias (1 male and 6 female), 3 Lis (all female), 1 Mulam (female), 1 Gelao (female), and 3 Xibes (2 male and 1 female). Informed consent was obtained from each participant. The flowchart is shown in Fig. 1.

### Anthropometric measurement

Participants came to the test site refraining from exercise and alcohol or stimulant beverages and fasting for at least 2 hours, and were requested to wear light clothes and no hats or shoes, then their body weight and height were measured using standardized instruments. Body weight was measured to the nearest 0.1 kg and height to the nearest 0.5 cm according to the standard procedures.

Body composition, including total body water (TBW), fat mass (FM), protein mass (PM), and mineral content (MC) [12, 13], was estimated by bioelectrical impedance analysis method using the body composition analyzer BCA-1B (Tsinghua Tongfang, China). Measurement was performed by a trained professional, and all participants were requested to remove all metal accessories and remain standing upright steadily with bare feet on the analyzer.

### Physical fitness tests

The physical fitness tests were performed following the State Students Health Standards of China [14], which included body mass index (BMI), vital capacity (VC), sit and reach, standing long jump, pull-ups/crunches, 50-m sprint, and 800/1000-m run. Detailed information on each test has been reported elsewhere [1, 15].

#### Body mass index

BMI is an indirect measure of body fat, and it was calculated as body weight (in kilograms) divided by the square of height (in meters), shown as follows: BMI = weight (kg)/height (m)^2^.

#### Vital capacity

Vital capacity is defined as the total volume of air that can be displaced from the lungs by maximal expiratory effort, thus it evaluated the breathing capacity and talks about respiratory health. VC was measured using the WQS-8888 model apparatus (Shanghai Wanqing, China). College students were required to put their mouths into the blowpipe, stand before the apparatus, and hold the handle properly. Then, students pressed the device’s button, took a deep breath, and completely exhaled. The apparatus automatically calculated the maximal breathing capacity.

#### Sit and reach

The sit and reach test was conducted to assess low back flexibility. The participants sat barefoot on the test instrument with stretched legs and gradually reached forward as far as possible. The test was administered twice, and the better score was recorded.

#### Standing long jump

The standing long jump test was conducted to assess lower-limb explosive strength. The participants stood at the starting line and were requested to jump forward as far as they could. The distance was measured in meters from the starting line to the heel of the closest foot. The test was administered twice, and the better score was recorded.

#### Pull-up

The pull-up test was used to evaluate the upper-body muscular strength. The test was scored according to the number of pull-ups. The participants jumped up and pulled the bars with both hands. After standing still, participants pulled with both arms at the same time. All the male students performed the test.

#### Crunches

The crunches is an abdominal endurance training exercise commonly performed to assess abdominal muscle strength. The participants were instructed to lay on a mat with knees bent at 90°, raise their upper body, and touch their knees with their elbows. The number of crunches completed in 1 minute was recorded. All the female students performed the test.

#### 50-m sprint

A 50-m sprint test was conducted to evaluate the speed and explosive strength of students. When the investigator said “go,” the participants began the 50-m run. They finished the run as fast as they could. The time in minutes and seconds was recorded.

#### 800 / 1000-m run

The 800 / 1000-m run is a comprehensive sport that requires speed and endurance. The students stood at the starting line and were requested to complete the 800- or 1000-m test as fast as they could. The time in minutes and seconds was recorded. All the female students performed the 800-m run, and all male students performed the 1000-m run.

### Physical fitness score

According to the State Students Health Standards of China, physical fitness was measured by the scores of physical fitness tests, which are composed of three parts, including body shape score (BMI accounts for 15 points), body function score (VC accounts for 15 points), and physical quality score (sit and reach accounts for 10 points, standing long jump accounts for 10 points, pull-ups/crunches account for 10 points, 50-m sprint accounts for 20 points, and 800/1000-m run accounts for 20 points). For college students, physical fitness scores were calculated as the total values, which were summed by the weighted score of each participant. The formula was expressed as follows: total physical fitness score = body shape score + body function score + physical quality score = BMI score × 15% + VC score × 15% + (sit and reach score × 10% + standing long jump score × 10% + pull-ups/crunches score × 10% + 50-m sprint score × 20% + 800/1000-m run score × 20%) [14].

Generally, BMI values were divided into four groups. Moreover, based on the State Students Health Standards of China, the BMI ranges for male students, which are different from those of the World Health Organization, are as follows: underweight, < 17.8 kg/m^2^; normal weight, 17.9–23.9 kg/m^2^; overweight, 24.0–27.9 kg/m^2^; and obese, > 28.0 kg/m^2^. Additionally, for female students, BMI ranges are as follows: underweight, < 17.1 kg/m^2^; normal weight, 17.2–23.9 kg/m^2^; overweight, 24.0–27.9 kg/m^2^; and obese, > 28.0 kg/m^2^. College students with BMI indicating normal obtain 100 points, underweight and overweight 80 points, and obese 60 points [14]. All the grading standards of the physical fitness tests are shown in Table 1.

**Table 1:** Scoring standards of the physical fitness test [14]

Score	BMI		Vital capacity		Sit and reach		Standing long jump		Bent-leg sit-up		50-m sprint^a^		1000/800-m run^a^	
	(kg/m^2^)		(L)		(cm)		(cm)		/Pull up (times)		(s)		(s)	
	Male	Female	Male	Female	Male	Female	Male	Female	Male	Female	Male	Female	Male	Female
100	17.9–23.9	17.2–23.9	5.04	3.40	24.9	25.8	273	207	19	56	6.7	7.5	197	198
95	–	–	4.92	3.35	23.1	24.0	268	201	18	54	6.8	7.6	202	204
90	–	–	4.80	3.30	21.3	22.2	263	195	17	52	6.9	7.7	207	210
85	–	–	4.55	3.15	19.5	20.6	256	188	16	49	7.0	8.0	214	217
80	≤17.8	≤17.1	4.30	3.00	17.7	19.0	248	181	15	46	7.1	8.3	222	224
	24.0–27.9	24.0–27.9												
78	–	–	4.18	2.90	16.3	17.7	244	178	–	44	7.3	8.5	227	229
76	–	–	4.06	2.80	14.9	16.4	240	175	14	42	7.5	8.7	232	234
74	–	–	3.94	2.70	13.5	15.1	236	172	–	40	7.7	8.9	237	239
72	–	–	3.82	2.60	12.1	13.8	232	169	13	38	7.9	9.1	242	244
70	–	–	3.70	2.50	10.7	12.5	228	166	–	36	8.1	9.3	247	249
68	–	–	3.58	2.40	9.3	11.2	224	163	12	34	8.3	9.5	252	254
66	–	–	3.46	2.30	7.9	9.9	220	160	–	32	8.5	9.7	257	259
64	–	–	3.34	2.20	6.5	8.6	216	157	11	30	8.7	9.9	262	264
62	–	–	3.22	2.10	5.1	7.3	212	154	–	28	8.9	10.1	267	269
60	≥28.0	≥28.0	3.10	2.00	3.7	6.0	208	151	10	26	9.1	10.3	272	274
50	–	–	3.94	1.96	2.7	5.2	203	146	9	24	9.3	10.5	292	284
40	–	–	2.78	1.92	1.7	4.4	198	141	8	22	9.5	10.7	312	294
30	–	–	2.62	1.88	0.7	3.6	193	136	7	20	9.7	10.9	332	304
20	–	–	2.46	1.84	−0.3	2.8	188	131	6	18	9.9	11.1	352	314
10	–	–	2.30	1.80	−1.3	2.0	183	126	5	16	10.1	11.3	372	324

Pull up and 1000-m run are only for male students; Bent-leg sit-up and 800-m run are only for female students

^a^In this test, lower values (in seconds) indicate higher performance (in scores)

### Data analysis

To investigate the associations between body composition (x) and physical fitness score (y), linear regression analyses were conducted. Two regression models were created: (a) unadjusted model and (b) model adjusted for the age and ethnicity of the college students. All continuous variables are reported as mean and standard deviation ($\overline{x}$ ± *s*). An independent-sample *t*-test was conducted to compare the mean difference among gender groups. Percentages were used to describe the qualitative variables, and the difference between two sexes was compared using a chi-squared test. Statistical analysis was performed using SPSS version 22. All hypothesis tests were two-sided, and a *P* < 0.05 was considered statistically significant.

## Results

### Descriptive statistics

A total of 2291 students from Shenyang Medical College completed the body composition measurement and the physical fitness test. Compared with female students, male students demonstrated significantly higher height, weight, TBW, PM, MC, BMI, VC, standing long jump, and body function score but lower FM, sit and reach, 50-m sprint, body shape score, physical quality score, and total physical fitness score. There was no significant difference in the mean age between the male and female students. The descriptive characteristics of all parameters among the two groups are shown in Table 2.

**Table 2:** Descriptive characteristics of the participants

Characteristic	All (2291)	Male (815)	Female (1476)	*P*^a^
Age (years)	19.09 ± 0.82	19.10 ± 0.82	19.07 ± 0.83	0.336
Anthropometric data				
Height (cm)	167.44 ± 8.54	175.24 ± 6.46	163.14 ± 6.18	^<^0.001
Weight (kg)	61.94 ± 13.27	70.90 ± 13.94	56.99 ± 9.87	^<^0.001
TBW (kg)	34.12 ± 7.55	42.04 ± 6.27	29.75 ± 3.61	^<^0.001
FM (kg)	15.00 ± 6.40	13.23 ± 6.28	15.97 ± 6.25	^<^0.001
PM (kg)	9.62 ± 2.13	11.86 ± 1.77	8.39 ± 1.02	^<^0.001
MC (kg)	3.08 ± 0.55	3.66 ± 0.46	2.77 ± 0.26	^<^0.001
Physical fitness test data				
BMI (kg/m^2^)	21.98 ± 3.68	23.04 ± 4.03	21.39 ± 3.33	^<^0.001
VC (L)	3.86 ± 1.02	4.83 ± 0.85	3.33 ± 0.64	^<^0.001
Sit and reach (cm)	16.87 ± 6.52	14.68 ± 6.74	18.08 ± 6.07	^<^0.001
Standing long jump (cm)	190.63 ± 32.28	224.25 ± 24.62	172.06 ± 17.68	^<^0.001
Pull-up (times)	–	4.53 ± 4.97	–	–
Bent-leg sit-up (times)	–	–	32.56 ± 9.50	–
50-m sprint (s)	8.50 ± 1.06	7.51 ± 0.74	9.05 ± 0.77	^<^0.001
1000-m run (s)	–	259.34 ± 36.14	–	–
800-m run (s)	–	–	240.56 ± 45.11	–
Physical fitness score				
Body shape score^b^	13.95 ± 1.81	13.52 ± 2.07	14.19 ± 1.61	^<^0.001
Body function score^b^	13.08 ± 1.94	13.20 ± 1.88	13.01 ± 1.97	0.02
physical quality score^b^	47.14 ± 7.29	43.85 ± 8.20	48.96 ± 6.01	^<^0.001
Total score of physical fitness^b^	74.17 ± 8.21	70.57 ± 9.22	76.16 ± 6.83	^<^0.001

Data are means±standard deviation

*TBW* total body water, *FM* fat mass, *PM* protein mass, *MC* mineral mass, *BMI* body mass index, *VC* vital capacity

^a^Refers to the *p* value of an independent test (continuous variables) between male and female students

^b^Means higher value (in scores) indicate better performance

### Association between the body composition and the physical fitness score

The associations between body composition (x) and physical fitness score (y) are reported in Tables 3 and 4. Only FM was negatively associated with body shape, body function, physical quality, and total physical fitness scores, and other body composition variables had no statistically significant associations with any physical fitness score (see Table 3). In the adjusted analyses, each unit (kg) increase in FM was associated with a 0.24-point (*P* < 0.001) decrease in the body shape score, a 0.04-point (*P* = 0.006) decrease in the physical function score, a 0.87-point (*P* < 0.001) decrease in the physical quality score, and a 1.15-point (*P* < 0.001) decrease in the total physical fitness score.

**Table 3:** Associations of body composition with physical fitness in male students^a^

	Body composition measures (x)											
	TBW (kg)			FM (kg)			PM (kg)			MC (kg)		
Physical fitness score (y)	b	SE	*P*	b	SE	*P*	b	SE	*P*	b	SE	*P*
Body shape score												
Unadjusted	−0.818	0.625	0.191	−0.236	0.012	***<*** **0.001**	−0.139	1.527	0.927	11.472	7.133	0.108
Adjusted^b^	−0.878	0.624	0.160	−0.241	0.012	***<*** **0.001**	0.241	1.522	0.874	10.880	7.110	0.126
Body function score												
Unadjusted	−0.411	0.804	0.609	−0.038	0.015	**0.011**	1.477	1.963	0.452	2.025	9.168	0.825
Adjusted^b^	−0.389	0.806	0.630	−0.042	0.015	**0.006**	1.706	1.965	0.386	0.861	9.180	0.925
Physical quality score												
Unadjusted	−1.553	3.353	0.643	−0.851	0.063	***<*** **0.001**	8.968	1.936	0.274	−9.470	38.240	0.804
Adjusted^b^	−1.661	3.363	0.621	−0.867	0.064	***<*** **0.001**	10.052	8.203	0.221	−12.034	38.320	0.754
Total score of physical fitness												
Unadjusted	−2.783	3.563	0.435	−1.125	0.067	***<*** **0.001**	10.306	8.701	0.237	4.028	40.634	0.921
Adjusted^b^	−2.928	3.562	0.411	−1.150	0.067	***<*** **0.001**	11.999	8.689	0.168	−0.292	40.591	0.994

*TBW* total body water, *FM* fat mass, *PM* protein mass, *MC* mineral mass, *x* independent variables, *y* dependent variables

^a^Analyzed using regression analysis. The unstandardized regression coefficient (b) with its standard error (SE) and the *P* value (*P*) are given for each association

^b^All models were adjusted for student’s age and ethnicity

**Table 4:** Associations of body composition with physical fitness in female students^a^

	Body composition measures (x)											
	TBW (kg)			FM (kg)			PM (kg)			MC (kg)		
Physical fitness score (y)	b	SE	*P*	b	SE	*P*	b	SE	*P*	b	SE	*P*
Body shape score												
Unadjusted	0.347	0.458	0.448	− 0.120	0.007	**< 0.001**	−1.178	1.073	0.272	−1.483	5.713	0.795
Adjusted^b^	0.358	0.457	0.433	−0.120	0.007	**< 0.001**	−1.130	1.073	0.292	−1.822	5.716	0.750
Body function score												
Unadjusted	−0.211	0.685	0.758	−0.019	0.010	0.061	1.621	1.607	0.313	−0.921	8.557	0.914
Adjusted ^b^	−0.200	0.685	0.770	−0.018	0.010	0.070	1.758	1.607	0.274	−1.613	8.560	0.851
Physical quality score												
Unadjusted	0.933	2.136	0.662	−0.228	0.032	**< 0.001**	−6.172	5.008	0.218	11.941	26.671	0.654
Adjusted ^b^	0.960	2.126	0.652	−0.225	0.031	**< 0.001**	−5.555	4.989	0.266	9.137	26.577	0.731
Total score of physical fitness												
Unadjusted	1.069	2.376	0.653	−0.367	0.035	**< 0.001**	−5.729	5.572	0.304	9.537	29.675	0.748
Adjusted ^b^	1.118	2.365	0.636	−0.363	0.035	**< 0.001**	−4.928	5.55	0.375	5.702	29.561	0.847

*TBW* total body water, *FM* fat mass, *PM* protein mass, *MC* mineral mass, *x* independent variables, *y* dependent variables

^a^Analyzed using regression analysis. The unstandardized regression coefficient (b) with its standard error (SE) and the *p* value (*p*) are given for each association

^b^All models were adjusted for student’s age and ethnicity

In female students, FM was associated with worse performance in the body shape, physical quality, and total physical fitness scores (see Table 4). More specifically, in the adjusted analyses, each unit (kg) increase in FM was associated with worse performance in the body shape score (− 0.12 points, *P* < 0.001), physical quality score (− 0.23 points, *P* < 0.001), and total physical fitness score (− 0.36 points, *P* < 0.001).

### Weight status in students according to sex

**Fig. 2:**
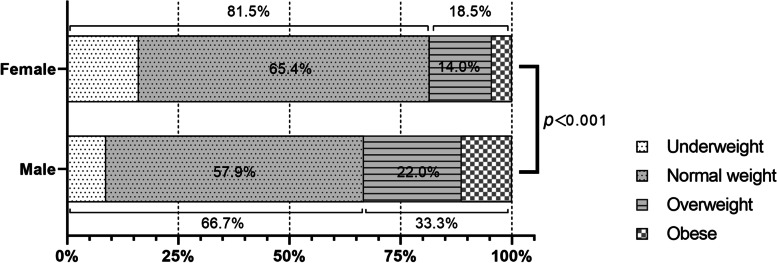
The constituent ration of weight status between the sexes

According to Chinese college students’ growth characteristics for age, sex, and BMI, the participants were classified as obese, overweight, normal weight, or underweight (Fig. 2). The ratio of overweight to obesity in male students was significantly higher than the ratio in female students. This seems to explain why the physical fitness performance of female students is usually much better than that of male students.

## Discussion

A two-component model of body composition divides the body into FM and fat-free mass (including TBW, PM, and MC) [12, 13]. FM is often closely related to the occurrence and development of cardiovascular diseases, diabetes, and other chronic diseases and the physical fitness status of the body [9, 16, 17]. Therefore, decreasing the proportion of FM in body composition is the first condition to remain healthy. Consistent with the results reported by other scholars [18, 19], this study indicated that there were gender differences in various body composition indexes of Shenyang multi-ethnic medical students, in which the TBW, PM and MC of female students were lower than those of male students except for FM. This phenomenon is mainly caused by genetic factors closely related to physiological characteristics. In adolescent girls, the estrogen level increases, which in turn promotes the accumulation of adipose tissue in the body [20], whereas in boys, whose body composition is mainly characterized by the increase of fat-free body weight, the androgen level increases [21].

At present, colleges and universities in China collect test data on students’ physical fitness based on the test items stipulated in the State Students Health Standards of China, and then they convert the test results into percentile scores according to the scoring standards to evaluate the physical fitness status of college students. The evaluation of the physical fitness status of college students in China is mainly carried out through three aspects: body shape, body function, and physical quality. Based on the results, the overall level of physical fitness of medical students in Shenyang is similar to that of college students in other cities in China [4, 22]. However, in terms of physical function, the score of male students is higher than that of female students; this result is the same as that of other research [23]. It was found that genetic factors and lifestyle may explain differences in vital capacity within sexes [15]. In addition, it is worth noting that the body shape, physical quality, and total physical fitness scores of female medical students are significantly higher than those of male students, which indicates that, to some extent, the physical health level of female students is generally better than that of male students. This is possibly influenced by social and psychological factors and professional knowledge, as female medical students generally pay more attention to good physique and health [24]. Therefore, a healthy lifestyle such as not smoking, not drinking, not staying up late, and not overeating helps female students maintain appropriate body shape and improve their physical fitness [1, 25].

It has been proved that BMI is associated with the physical fitness status of college students [1, 26]. Although BMI is correlated with the FM of the population, the fat mass of the body changes with age, and the rate of this change varies according to sex, race, and individual differences, indicating that BMI alone is not sensitive to body composition [27]. For this reason, this paper further discusses the relationship between body composition and physical fitness in medical students. First, body shape of Chinese college students can be expressed by the level of scores, and the students with higher BMI obtain lower scores according to the scoring standards. Therefore, overweight and obese students can inevitably get lower scores in terms of body shape. Second, some studies have confirmed that there is a significant correlation between higher FM and lower VC [15, 28]. This result also supports the aforementioned conclusion that there is a negative correlation between FM and the score of body function (VC) only in male students. The reasons are as follows: firstly, the increase of FM in the chest and abdominal cavity leads to a decrease in pulmonary compliance; at the same time, the respiratory muscle and trachea are squeezed and deformed, limiting the ventilation capacity of the lung tissue [29]. Secondly, endocrine, glucose, and lipid metabolism disorders induced by the accumulation of adipose tissue indirectly affect pulmonary ventilation [30, 31]. What needs to be clarified is that there were no significant association between FM and the score of body function in female students, although the *P* value was close to 0.05 in statistical significance testing. We speculate that this result may be closely related to the differences in fat distribution between the two sexes, as Huang et al. pointed out that the sensitivity of associations between fat distribution and lung function is different for both sexes [15].

In addition, some scholars have found that being overweight or obese can reduce the scores in different test items of sit and reach, standing long jump, pull-ups/crunches, 50-m sprint, and 800/1000-m run to a certain extent [1, 31]. In this study, we converted the test scores of all above items into a total score following the scoring standards, which are used to reflect the overall level of physical quality of participants. We found that FM also has an adverse effect on the students’ physical quality. When the body FM increases excessively, first, the physical burden increases, and then, the exercise ability decreases [32]. Second, the accumulation of abdominal fat inevitably limits waist movement, such as difficulty in bending forward [33]. Third, after the accumulation of fat in skeletal muscle and surface, muscle contraction will more or less hinder the performance of endurance, flexibility, agility, speed, and other qualities [34]. Fourth, people who are overweight and obese generally have lower levels of gas exchange and oxygen utilization and are prone to an insufficient oxygen supply, resulting in a decline in endurance exercise ability [35].

It should also be pointed out that the comparison of BMI composition between the two sexes shows that male medical students have a higher rate of overweight and obesity, indicating that the state of their physical fitness is more alarming [36].

There are some limitations to the study that should be considered. Firstly, although the number of medical students in this study is enough, the sample could not truly represent the entire medical students in China, as most of the participants were from Shenyang city in Liaoning Province. Secondly, this study is a cross-sectional study which findings suggest that FM is associated with physical fitness, but the cause-and-effect relationship has not been established. Thirdly, our findings neglected the effects of FM in different parts of the body on physical fitness. In other words, we cannot confidently extrapolate findings to how body fat distribution affects physical fitness of Chinese medical students.

This study provides initial evidence of Chinese medical students’ body composition and physical fitness, which may have implications to further research. Based on the above findings, the whole society should pay more attention to the physical fitness of medical students, and colleges and universities should also strengthen to guide students to exercise more scientifically, which is the best way to improve physical fitness for medical students.

## Conclusions

To sum up, in this study, a higher FM was significantly associated with worse physical fitness among medical students of Shenyang in China, and the physical fitness level of female students is better than that of male students. Moreover, male students with a higher rate of overweight and obesity are an important group that needs weight control.

## Data Availability

The datasets generated and/or analyzed during the current study are not publicly available due to limitations of ethical approval involving the student data and anonymity but are available from the corresponding author on reasonable request.

## Abbreviations

BMI: Body mass index
FM: Fat mass
MC: Mineral content
PM: Protein mass
TBW: Total body water
VC: Vital capacity
